# Randomized Evaluation of Videoconference Meetings for Medical Students’ Mid-clerkship Feedback Sessions

**DOI:** 10.5811/westjem.2018.10.39641

**Published:** 2018-11-26

**Authors:** Zhengqiu Zhou, Theresa Mims, Adam Dugan, Terren Trott, William Sanderson, Jonathan Bronner

**Affiliations:** * University of Kentucky, Department of Emergency Medicine, Lexington, Kentucky; † Skagit Valley Hospital, Department of Emergency Medicine, Mount Vernon, Washington

## Abstract

**Introduction:**

Videoconferencing has been employed in numerous medical education settings ranging from remote supervision of medical trainees to conducting residency interviews. However, no studies have yet documented the utility of and student response to videoconference meetings for mid-clerkship feedback (MCF) sessions required by the Liaison Committee on Medical Education (LCME).

**Methods:**

From March 2017 to June 2018, third-year medical students rotating through the mandatory, four-week emergency medicine (EM) clerkship at a single medical school were randomly assigned either to a web-based videoconference meeting via Google Hangouts, or to a traditional in-person meeting for their MCF session. To compare students’ MCF experiences we sent out an electronic survey afterward to assess the following using a 0–100 sliding scale: overall satisfaction with the meeting; the effectiveness of communication; the helpfulness of the meeting; their stress levels, and the convenience of their meeting location. The survey also collected data on these demographic variables: the name of the faculty member with whom the student met; student gender, age, and interest in EM; location prior to meeting; meeting-method preference; and number of EM shifts completed.

**Results:**

During the study period, 133 third-year medical students responded to the survey. When comparing survey responses between individuals who met online and in person, we did not detect a difference in demographics with the exception of preferred meeting method (p=0.0225). We found no significant differences in the overall experience, helpfulness of the meeting, or stress levels of the meeting between those who met via videoconference vs. in-person (p=0.9909; p=0.8420; p=0.2352, respectively). However, individuals who met in-person with a faculty member rated effectiveness of communication higher than those who met via videoconference (p=0.0002), while those who met online rated convenience higher than those who met in-person (p<0.0001). Both effects remained significant after controlling for preferred meeting method (p<0.0001 and p=0.0003, respectively) and among EM-bound students (p=.0423 and p<0.0110, respectively).

**Conclusion:**

Our results suggest that LCME-required MCF sessions can be successfully conducted via web-based programs such as Google Hangouts without jeopardizing overall meeting experience. While the convenience of the meetings was improved, it is also important for clerkship directors to note the perceived deficit in the effectiveness of communication with videoconferencing.

## INTRODUCTION

Mid-clerkship feedback (MCF) sessions are formal, one-on-one meetings between medical students and faculty members to assess student progress and address any remediation needs. It is a Liaison Committee on Medical Education (LCME) requirement for all medical school clerkships of four weeks or more in duration.^1^ These meetings are traditionally completed in-person; however, it is not uncommon for scheduling difficulties to arise for rotations with varying schedules such as emergency medicine (EM). With advancements in technology, videoconferencing has become widely available and could potentially provide a solution to this problem.

Videoconferencing has been shown to be beneficial in various medical education^2^,^3^ and inpatient care settings.^4^–^6^ Cameron et al.^5^ and Xavier et al.^6^ assessed the use of videoconferencing for the supervision and training of medical professionals, and both studies found that the majority of participants rated use of technology as “positive.” Bertsch et al.^2^ and Stain et al.^3^ found that material delivered via online lectures was as effective as traditional in-person lectures. Other studies have also reported improved convenience with online interviews for conducting residency^7^ and fellowship interviews.^8^ While videoconference use has been described in these medical or educational settings, the use of videoconferencing for MCF meetings has not yet been investigated. We conducted a prospective, randomized controlled study to examine the utility of and student response to using videoconference for MCF sessions. Based on previously documented, successful utilization of videoconferencing, we hypothesized that videoconferencing could be as effective as in-person meetings for MCF sessions.

## METHODS

All third-year medical students who rotated through our EM clerkship between March 2017 and June 2018 were invited to participate in the study. During each four-week rotation, 8–12 students participated in the EM clerkship. Individuals who consented to participate in the study were randomly assigned to either a web-based videoconference meeting or a traditional in-person meeting for their EM mid-clerkship feedback session by block randomization. The MCF sessions were conducted by one of three EM faculty members. Each student was assigned to a meeting time with a faculty member based on the faculty member’s availability. All meetings were scheduled for 30 minutes during standard business hours. If their meeting time coincided with a shift, the students were excused from their shift for the duration of the meeting. The meeting involved a case presentation by the student, a review of current clinical grades, and a discussion of the student’s strengths and weaknesses. In-person meetings were held in the EM faculty member’s office. For videoconference meetings, faculty remained in their offices, whereas students were informed they could access their meeting from anywhere with reliable Internet access. Google Hangouts (Mountain View, California) was used as the videoconference platform.

**Population Health Research Capsule**

What do we already know about this issue?

*Videoconferencing is utilized successfully in various medical education settings, though its use for medical student Mid-Clerkship Feedback (MCF) sessions has not yet been investigated*.

What was the research question?

*We investigated student perceptions on the use of videoconferencing for mandatory MCF meetings between third-year medical students and our emergency medicine faculty*.

What was the major finding of the study?

*MCF via videoconference can be successful without jeopardizing overall experience. However, perceived communication was rated lower and convenience higher than those that met in-person*.

How does this improve population health?

*Our finding provides evidence that clerkship directors can potentially incorporate videoconferencing for MCF meetings as an option depending on students’ needs*.

After their meeting, the participants were invited via university email to complete an anonymous electronic survey (Supplemental Figure) to assess their meeting experience. The survey asked students to rate five aspects of their meeting – overall experience, effectiveness of communication, helpfulness of meeting for their learning, stress levels during their meeting, and convenience of the meeting location, on a sliding scale from 0–100. We designed the first question to assess the student’s overall satisfaction with their meeting experience (overall experience). The following four questions were designed to help understand the factors that may have influenced their overall experience (communication, helpfulness, stress levels, and convenience). These four factors were identified by faculty and medical students as important determinants for a successful meeting in the setting of mid-clerkship formative feedback.

We used a 0–100 scale since it provided students with greater flexibility, and it would result in greater statistical power compared to an ordinal scale. The directionality of the scale was indicated on the survey as seen in Supplemental Figure. The survey question, “How was your overall experience with Mid-Clerkship Feedback session?” will be henceforth referred to as “overall experience.” The survey question, “How effective was the communication using your meeting modality?” will be referred to as “communication.” The survey question, “How was your stress level during the meeting?” will be referred to as “stress levels.” The survey question, “How convenient was the meeting location for you?” will be referred to as “convenience.”

The survey also included questions regarding the following factors: faculty with whom they met, student’s gender and age, shifts completed prior to meeting, interest in EM, location prior to meeting, and meeting-method preference. To maintain confidentiality, the names of the faculty members from the survey have been removed in Supplemental Figure. Doctors “X,” “Y,” and “Z” have been used in place of their names. Answers to all survey questions were required except for the free-text answer to “Additional suggestions for how to improve mid-clerkship feedback sessions?” A reminder email was sent every two days up to a maximum of five times, or until completion of the survey. We collected and managed the survey data using the Research Electronic Data Capture (REDCap)^9^ tool hosted at our home institution. The study was reviewed and approved by our local institutional review board.

### Statistical Analysis

Student variables were stratified by meeting method for analysis. For categorical variables, frequencies and column percentages were reported. We calculated p-values using chi-square and Fisher’s exact tests, as appropriate to determine statistical significance. For normally distributed continuous variables, we reported means and standard deviations, and we calculated p-values using t-tests; otherwise, medians and 25^th^/75^th^ percentiles were reported, and p-values were calculated using Mann-Whitney U tests. We used multivariable linear regression models to adjust for potential confounding variables in the relationship between student ratings and group assignment. All analyses were done in R programming language, version 3.5.0 (R Foundation for Statistical Computing, Vienna, Austria). Statistical significance was set to a p<0.05.

## RESULTS

Of the 163 third-year medical students who rotated through EM during the research period, 141 consented to participate in the study (86.5%). Eight of the 141 students (5.6%) were excluded from the study prior to the completion of their survey. Five of the eight were excluded due to scheduling conflicts that resulted in a change in their assigned meeting method; one student withdrew from the study due to a personal preference for the alternative meeting method; one was unable to meet online due to technical difficulties; and one was excluded from the study due to a leave of absence from medical school. Of the 133 remaining participants, the survey completion rate was 100%. Sixty-seven participants were randomized to the videoconference group and 66 participants were randomized to the in-person group.

Demographic variables are detailed in Table 1. Dr. X met with the largest portion of students for their MCF meetings (57.1%). The majority of the participants (56.4%) were between 25–29 years old, male (54.1%), expressed that they did not have any interest in EM as a future career choice (57.9%), and listed their location prior to meeting as “home” (55.6%). The participants had completed an average of seven shifts prior to their mid-clerkship meetings. When comparing those who met online vs. in-person, the only demographic variable that significantly differed between the two groups was their preferred meeting method (p=0.0225). Of those students who did meet online, a significantly higher proportion of them reported a preference to meeting online (46.3% videoconference group vs 24.2% in-person group). Among those who met in-person, a greater proportion of students reported preferring to meet in-person (56.1% in-person group vs. 35.8% videoconference group). In the response to the survey question, “Additional suggestions for how to improve MCF sessions,” we identified several common themes. Seven of the 67 individuals who met via videoconference reported some degree of technical difficulty, three of the 67 suggested allowing students to choose their meeting method, and two students commented on the difficulty of finding an appropriate location for a videoconference while on campus. Of those students who met in-person, one of the 66 also suggested allowing students to self-select the meeting method.

To determine if there were differences in experience between those who met online vs. in-person, we compared participants’ sliding scale ratings of overall experience, communication, helpfulness, stress levels and convenience of meeting location between the two groups (Figure, Table 2). We found no significant differences in the scores between videoconference and in-person meetings in overall experience, helpfulness of meeting, stress levels, or convenience. (Median overall experience score: 90.0 for videoconference, 91.5 for in-person, p=0.9909; median helpfulness score: 80.0 for videoconference, 85.0 for in-person, p=0.8420; median stress level score: 20.0 for videoconference, 22.5 for in-person, p=0.2352.) However, individuals meeting in-person rated effectiveness of communication higher than those meeting via videoconference (median score: 85.0 for videoconference, 100 for in-person, p=0.0002), but with significantly lower convenience (median score: 100 for videoconference, 75.0 for in-person, p<0.0001).

Since preferred meeting method was found to differ significantly between the two groups, we used multivariable linear regression models to control for its effect on the relationship between study group and the rating score (Supplemental Table 1). Overall, the results did not change after controlling for preferred meeting method. Overall meeting satisfaction, helpfulness of the meeting, and stress levels during the meeting still did not differ significantly between the groups (p=0.9680, p=0.8650, and p=0.6615, respectively). Effectiveness of communication and convenience of meeting location were still found to differ significantly between the study groups; participants who met in-person rated communication higher than those who met online. (Mean difference [MD]: 13.9, 95% confidence interval [CI] [7.724–20.108], p<0.0001) and videoconference group rated convenience higher than those who met in-person (MD [−16.817], 95% CI [−25.802 – −7.833]), p=0.0003).

To assess the impact of videoconferencing on students interested in a career in EM, we completed a subgroup analysis on the 31 participants who selected “Yes” to the question, “Do you have an interest in EM career?” Among the demographic variables, no significant difference was identified between individuals meeting via videoconference compared to in-person in any of the variables including preferred meeting method (p=0.0688, Supplemental Table 2). Since these results were borderline significant, we hypothesize that with a larger sample size, significance may also have been achieved. The results of the rating scales in students interested in EM were the same as the overall results. Effectiveness of communication and convenience of meeting remained significantly different (p=0.0423 and p=.0110, respectively), whereas no significant differences were observed in student ratings for overall experience, helpfulness, and stress levels (p=0.7102, p=0.1520, and p=0.8731, respectively) (Supplemental Table 3).

## DISCUSSION

Our study assessed the use of videoconference for medical students’ MCF sessions using a prospective, randomized controlled study. We found no difference in overall experience, stress levels and helpfulness of the meeting between students who met via videoconference compared to those who met in-person. However, we did identify significant differences in convenience and communication between the two groups. The non-significant, equally high ratings of overall experience in all participants support our hypothesis that the videoconference can be as effective as in-person meetings for MCF sessions. Our randomized controlled study design provides solid evidence for the non-inferiority of videoconference, indicating that the use of videoconference is a viable option for MCF meetings. However, it is important for faculty and students to note that, while videoconferencing improved convenience, as it can be conducted from any location with Internet access, students felt it jeopardized their communication capabilities. Hence, our study has helped faculty and students identify these as important factors to consider when selecting a meeting method. Additionally, as suggested in students’ qualitative comments, providing them a choice of preferred meeting method may be the optimal solution. Students may have personal preferences regarding which qualities they valued more. Additional studies are needed to confirm whether using meeting methods consistent with student preference has additional benefits on meeting satisfaction as, outside the context of a research study, it is unlikely that students would be randomized to a meeting method.

We suspect that the lack of improved overall experience despite higher convenience may be due to the hindrances in communication experienced by the participants. In addition to the lower communication ratings in the videoconference group, seven videoconference participants indicated in the free-text response section that they experienced some level of technical difficulty during their meetings, and one student was unable to complete a meeting due to technological issues. We did not include a question on our survey that specifically asked about technical challenges, as we wanted to keep the survey identical between the two groups. Therefore, it may be possible that additional students experienced technical difficulties but did not mention them in the “additional suggestions” section. In this regard, further investigation of the quality of communication problems would lend insight to future implementation of videoconferencing for MCFs. As technology continues to advance, we expect the audiovisual quality and Internet speeds to concurrently improve and lead to globally enhanced communication capabilities. Lastly, several other studies have used multiple platforms for online medical education, such as FaceTime and Skype.^10^,^11^ Assessing multiple videoconferencing platform options would also be beneficial for future studies to identify the characteristics of the electronic platform that are best suited to the study population.

As expected based on previous literature,^7^,^8^ convenience was improved with online meetings. Although not statistically significant, more people were at home immediately before their meetings (61.2%) in the videoconference group than the in-person group (50.0%). We can most likely attribute the difference in improved convenience to people not having to leave the comforts of home for their meetings. Of the five students who were dropped from the study due to a change in meeting method, three switched from meeting in-person to meeting online due to unexpected weather conditions that resulted in the closure of the university. Although survey data were not obtained from those three individuals, convenience may have been improved for these individuals as well. While our study does not include multiple campuses, we anticipate that not having to travel would further improve meeting satisfaction and convenience for students at satellite locations who need to complete their MCFs.

We are pleased to show that there was no difference in student ratings of their stress levels and perceived helpfulness of their MCF meetings. These results provide additional evidence for the non-inferiority of using videoconference for MCF meetings. It is important to note that stress levels were equally low in both groups of students, indicating MCF meetings at our institution are carried out under low-intensity conditions.

Faculty experience was not formally assessed in this study; however, the general feedback from all three attending physicians who participated in the study was positive. The faculty reported that the videoconference format allowed the concurrent completion of the institution’s electronic evaluation forms, which improved the efficiency of the meetings and the perceived accuracy of the evaluation forms. A drawback to videoconferences reported by the faculty was that several students required extra time to obtain video and audio function or to download plug-ins. However, similar delays also occurred with the in-person meetings due to tardiness. The exact number of individuals and the exact durations of the delays were not formally documented in our study. Formal assessments of faculty experience are encouraged in future studies.

## LIMITATIONS

There are several limitations to our study. First, eight students were excluded from the study after randomization because they elected to meet via the alternative meeting method. We are aware that this may have eliminated several undesirable or desirable ratings of the particular meeting method. Similarly, there was also a minority of students that rescheduled their meeting times based on the meeting method they were randomized into. For instance, if a student had been randomized to do an in-person meeting on a day after an overnight shift, the student may have elected to email the faculty member to ask if they could meet on an alternative day to avoid having to come to campus on post-call day. The faculty members made alternative arrangements whenever possible. Rescheduling for a more convenient time may also have affected survey ratings.

Additionally, there may have been biases in the population that consented to participate in the study. It is highly likely that those individuals with strong preferences on particular meeting method deferred the study and self-selected the preferred meeting method; therefore, their experiences are not captured. However, the highly significant differences in meeting convenience and communication despite controlling for the preferred meeting method suggest that a student’s preference for a particular meeting method did not affect the overall results of our study.

Lastly, with regard to the study’s methodology the survey instrument has not been previously validated. Although the survey questions do not satisfy any of the “common pitfalls of survey design” detailed by Artino et al.^12^, the wording of the questions, directionality of sliding scale, and the layout of the electronic survey could have influenced our results. We also note that this was a single-center study, which may limit the generalizability of our results.

## CONCLUSION

Our study provides preliminary evidence for the efficacy of videoconferences for routine meetings between faculty and medical students during MCF sessions. The survey data showed no differences in the overall experience of individuals meeting via videoconference compared to in-person. Given the improved convenience of videoconferencing, it may be beneficial for clerkship directors to provide it as a meeting option to provide more flexibility for students. However, it is also important for faculty to be aware of the perceived decrease in communication effectiveness related to videoconferencing along with the possibility of technical difficulties. Additional studies in multiple academic locations and using better-validated study tools are needed to confirm the results of our study.

## Supplementary Material









## Figures and Tables

**Figure:**
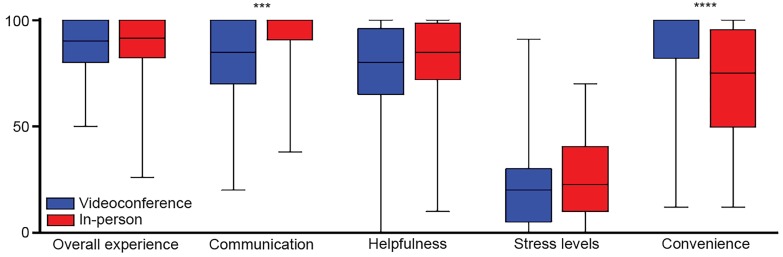
Box and whiskers plot comparing participant ratings. Statistical analysis conducted using t-test or Mann-Whitney U as appropriate. Displayed is median, interquartile range and minimum to maximum. ***p<0.001. ****p<0.0001.

**Table 1:** Analyses of study participants’ demographic variables comparing individuals meeting via videoconference vs. in-person for their mid-clerkship feedback sessions.

Variable	Overall	Videoconference	In-person	P value*
Number of students	133	67	66	
Faculty member, N (%)				
Dr. X	76 (57.1%)	35 (52.2%)	41 (62.1%)	0.5227
Dr. Y	47 (35.3%)	27 (40.3%)	20 (30.3%)	0.5227
Dr. Z	10 (7.5%)	5 (7.5%)	5 (7.6%)	0.5227
Student gender, N (%)				
Female	61 (45.9%)	32 (47.8%)	29 (43.9%)	0.7885
Male	72 (54.1%)	35 (52.2%)	37 (56.1%)	0.7885
Student age, years, N (%)				
20 – 24	42 (31.6%)	20 (29.9%)	22 (33.3%)	0.8390
25 – 29	75 (56.4%)	38 (56.7%)	37 (56.1%)	0.8390
30 +	16 (12.0%)	9 (13.4%)	7 (10.6%)	0.8390
Student interest in EM career, N (%)				
No	77 (57.9%)	40 (59.7%)	37 (56.1%)	0.4966
Undecided	25 (18.8%)	10 (14.9%)	15 (22.7%)	0.4966
Yes	31 (23.3%)	17 (25.4%)	14 (21.2%)	0.4966
Location immediately prior to meeting, N (%)				
Home	74 (55.6%)	41 (61.2%)	33 (50.0%)	0.3770
Campus	23 (17.3%)	10 (14.9%)	13 (19.7%)	0.3770
ED shift	35 (26.3%)	15 (22.4%)	20 (30.3%)	0.3770
Other	1 (0.8%)	1 (1.5%)	0 (0.0%)	0.3770
Preferred meeting method, N (%)				
In-person	61 (45.9%)	24 (35.8%)	37 (56.1%)	0.0225
Online	47 (35.3%)	31 (46.3%)	16 (24.2%)	0.0225
No preference	25 (18.8%)	12 (17.9%)	13 (19.7%)	0.0225
EM shifts completed, median (25th – 75th percentile)	7.0 (6.0 – 8.0)	7.0 (6.0 – 8.0)	7.0 (6.0 – 8.0)	0.5317

*EM*, emergency medicine; *ED*, emergency department.

* Video conference versus in-person.

**Table 2:** Comparison of participant ratings in individuals randomized to videoconference compared to in-person meetings for their mid-clerkship feedback sessions.

Variable	Videoconference median (25th – 75th percentiles)	In-person median (25th – 75th percentiles)	P-value videoconference versus in-person
Number of students	67	66	
Overall experience	90.0 (80.0 – 100.0)	91.5 (83.3 – 100.0)	0.9909
Communication	85.0 (72.5 – 100.0)	100.0 (91.3 – 100.0)	0.0002
Helpfulness	80.0 (67.0 – 96.0)	85.0 (72.3 – 97.5)	0.8420
Stress levels	20.0 (7.0 – 29.5)	22.5 (10.0 – 40.0)	0.2352
Convenience	100.0 (83.5 – 100.0)	75.0 (50.0 – 95.0)	<0.0001
