# Supplementary material for: Randomized Evaluation of Videoconference Meetings for Medical Students’ Mid-clerkship Feedback Sessions
Source: West J Emerg Med. 2018 Nov 26;20(1):163–9. doi: 10.5811/westjem.2018.10.39641 (PMC6324714; doi:10.5811/westjem.2018.10.39641)
Supplement: Supplementary file 1 [file wjem-20-163-s001.docx]

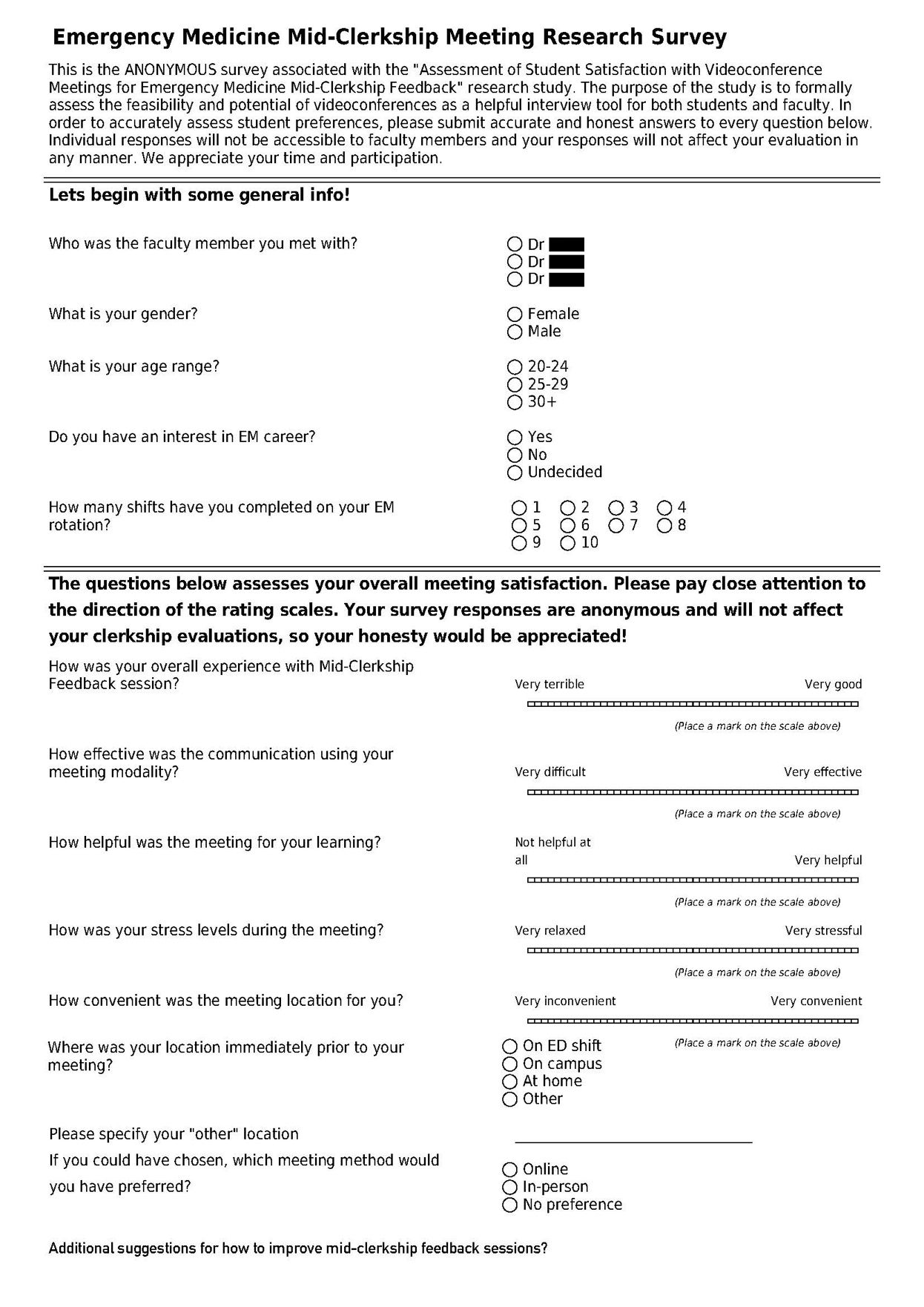


**Supplementary Figure.** Representation of electronic survey emailed to participants to assess for their experience during mid-clerkship feedback sessions.
