# Supplementary material for: Randomized Evaluation of Videoconference Meetings for Medical Students’ Mid-clerkship Feedback Sessions
Source: West J Emerg Med. 2018 Nov 26;20(1):163–9. doi: 10.5811/westjem.2018.10.39641 (PMC6324714; doi:10.5811/westjem.2018.10.39641)
Supplement: Supplementary file 2 [file wjem-20-163-s002.docx]

**Supplemental Table 1.** The effect of in-person vs videoconference meetings on participants’ ratings after controlling for preferred meeting method using linear regression model analysis.

| Outcome variable | Average difference (95% CI),  in-person versus videoconference | P-value |
| --- | --- | --- |
| Overall experience | 0.105  (-5.020, 5.231) | 0.9680 |
| Communication | 13.916  (7.724, 20.108) | <0.0001 |
| Helpfulness | -0.670  (-8.438, 7.098) | 0.8650 |
| Stress levels | 1.616  (-5.671, 8.904) | 0.6615 |
| Convenience | -16.817  (-25.802, -7.833) | 0.0003 |
