# Supplementary material for: Randomized Evaluation of Videoconference Meetings for Medical Students’ Mid-clerkship Feedback Sessions
Source: West J Emerg Med. 2018 Nov 26;20(1):163–9. doi: 10.5811/westjem.2018.10.39641 (PMC6324714; doi:10.5811/westjem.2018.10.39641)
Supplement: Supplementary file 3 [file wjem-20-163-s003.docx]

**Supplemental Table 2.** Subgroup analysis for demographic variables of participants who were interested in emergency medicine (EM) as a career choice.

|  | Overall | Videoconference | In person | P-value  videoconference versus in-person |
| --- | --- | --- | --- | --- |
| Number of students | N=31 | N=17 | N=14 |  |
| Faculty member, N (%) |  |  |  | 0.6808 |
| Dr. X | 15 (48.4%) | 7 (41.2%) | 8 (57.1%) |  |
| Dr. Y | 13 (41.9%) | 8 (47.1%) | 5 (35.7%) |  |
| Dr. Z | 3 (9.7%) | 2 (11.8%) | 1 (7.1%) |  |
| Student gender, N (%) |  |  |  | 1.0000 |
| Female | 10 (32.3%) | 5 (29.4%) | 5 (35.7%) |  |
| Male | 21 (67.7%) | 12 (70.6%) | 9 (64.3%) |  |
| Student age, years, N (%) |  |  |  | 0.2870 |
| 20 – 24 | 12 (38.7%) | 5 (29.4%) | 7 (50.0%) |  |
| 25 – 29 | 16 (51.6%) | 9 (52.9%) | 7 (50.0%) |  |
| 30 + | 3 (9.7%) | 3 (17.6%) | 0 (0.0%) |  |
| Location immediately prior to meeting, N (%) |  |  |  | 0.0965 |
| Home | 13 (41.9%) | 10 (58.8%) | 3 (21.4%) |  |
| Campus | 8 (25.8%) | 3 (17.6%) | 5 (35.7%) |  |
| ED shift | 9 (29.0%) | 3 (17.6%) | 6 (42.9%) |  |
| Other | 1 (3.2%) | 1 (5.9%) | 0 (0.0%) |  |
| Preferred meeting method, N (%) |  |  |  | 0.0688 |
| In-person | 16 (51.6%) | 8 (47.1%) | 8 (57.1%) |  |
| Online | 7 (22.6%) | 2 (11.8%) | 5 (35.7%) |  |
| No preference | 8 (25.8%) | 7 (41.2%) | 1 (7.1%) |  |
| EM Shifts Completed, Median (25^th^ – 75^th^ Percentile.) | 7.0 (6.0 – 8.0) | 6.0 (6.0 – 8.0) | 7.0 (6.0 – 7.0) | 0.9838 |
