# Supplementary material for: Randomized Evaluation of Videoconference Meetings for Medical Students’ Mid-clerkship Feedback Sessions
Source: West J Emerg Med. 2018 Nov 26;20(1):163–9. doi: 10.5811/westjem.2018.10.39641 (PMC6324714; doi:10.5811/westjem.2018.10.39641)
Supplement: Supplementary file 4 [file wjem-20-163-s004.docx]

**Supplemental Table 3.** Subgroup analysis of participant ratings in individuals who were interested in emergency medicine as a career choice.

| Variable | Videoconference  median (25^th^ – 75^th^ percentiles) | In person  median (25^th^ – 75^th^ percentiles) | P-value  videoconference versus in-person |
| --- | --- | --- | --- |
| Number of students | N=17 | N=14 |  |
| Overall experience | 95.0  (73.0 – 100.0) | 95.5  (88.5 – 100.0) | 0.7102 |
| Communication | 86.0  (50.0 – 100.0) | 100.0  (93.8 – 100.0) | 0.0423 |
| Helpfulness | 80.0  (75.0 – 100.0) | 90.0  (81.3 – 98.5) | 0.1520 |
| Stress levels | 24.0  (10.0 – 30.0) | 20.0  (4.8 – 30.0) | 0.8731 |
| Convenience | 100.0  (100.0 – 100.0) | 85.5  (57.0 – 96.0) | 0.0110 |
